# Deep learning-assisted analysis of biomarker changes after increase of dosing from aflibercept 2 mg to 8 mg in therapy-resistant neovascular age-related macular degeneration

**DOI:** 10.1136/bmjophth-2025-002176

**Published:** 2025-06-01

**Authors:** Michael Hafner, Ben Asani, Franziska Eckardt, Caspar Liesenhoff, Alexander Kufner, Jakob Siedlecki, Benedikt Schworm, Siegfried Priglinger, Johannes Benedikt Schiefelbein

**Affiliations:** 1 Department of Ophthalmology, LMU University Hospital, LMU Munich, Munich, BY, Germany

**Keywords:** Macula, Macular Degeneration, Retina, Treatment Medical, Imaging

## Abstract

**Purpose:**

Age-related macular degeneration (AMD) remains the leading cause of blindness in developed countries. There are many different intravitreal anti-vascular endothelial growth factor (VEGF) drugs available for the treatment of neovascular AMD (nAMD). Unfortunately, not all patients respond equally well to the drugs, and some show recurrences during treatment. Since 01/2024, aflibercept 8 mg represents an additional treatment option and contains a four times higher dosage than the already known aflibercept 2 mg.

**Methods:**

To evaluate the real-world efficacy of aflibercept 8 mg in refractory nAMD patients, focusing on changes in key optical coherence tomography biomarkers over a follow-up period of the first four aflibercept 8 mg injections using a deep learning-based semantic segmentation algorithm. Inclusion criteria were: switch to aflibercept 8 mg after insufficient response to aflibercept 2 mg, marked by persistent retinal fluid or inability to extend treatment beyond 6 weeks; completion of at least 3 months (90 days) follow-up under treat-and-extend treatment regime; and no confounding conditions like intraocular infection, uveitis or other retinal diseases.

**Results:**

23 eyes of 21 patients with therapy-resistant nAMD were switched to aflibercept 8 mg. All patients had previously received aflibercept 2 mg, with an average of 30.7 previous anti-VEGF injections. Significant reductions in intraretinal fluid and fibrovascular pigment epithelial detachment at timepoint V3 were observed. The decrease in subretinal fluid and central retinal thickness at V3 was not significant. Treatment intervals extended significantly by 24%, from a baseline average of 34 days to 42 days. Best-corrected visual acuity remained stable throughout the study period.

**Conclusions:**

Aflibercept 8 mg demonstrated significant efficacy and durability in reducing nAMD biomarkers and extending intervals in a real-world setting. The use of deep learning for biomarker quantification highlighted its potential for enhancing treatment monitoring and decision-making. Future studies with a larger patient cohort and prospective study setting should explore long-term outcomes and integration of artificial intelligence-driven analysis.

**WHAT IS ALREADY KNOWN ON THE TOPIC**

- Intravitreal injections of aflibercept 8 mg were proven safe in studies and were not inferior to previous anti-vascular endothelial growth factor therapies.

**WHAT THIS STUDY ADDS**

- In this study, the use of aflibercept 8 mg was shown to be safe in a real-world setting in patients who had already received multiple prior intravitreal treatments and was able to reduce key biomarkers of disease activity and thus extend treatment intervals.

**HOW THIS STUDY MIGHT AFFECT RESEARCH, PRACTICE, AND POLICY**

- This study shows the need for additional studies to further improve the complex treatment of poor and non-responders in the future and shows that a switch to aflibercept 8 mg may be beneficial in neovascular age-related macular degeneration patients who are refractory to aflibercept 2 mg.

## Introduction

 Age-related macular degeneration (AMD) has become the primary cause of blindness in developed countries.^1^ The treatment of neovascular AMD (nAMD) primarily targets abnormal angiogenesis in the retina caused by choroidal and macular neovascularisation (CNV/MNV).^2^ Inhibition of vascular endothelial growth factor (VEGF) through intravitreal injections has become a well-established and highly effective approach to preventing MNV growth and exudation, thereby preserving retinal structure. This method has transformed the management of neovascular retinal diseases.^3^

Given the chronic nature of nAMD, which far exceeds the half-life of current anti-VEGF agents, patients require ongoing retreatment, with up to 24 injections needed in the first 2 years under a fixed monthly regimen.^3^ To reduce the treatment burden and acknowledge that MNV reactivation follows an individual pattern, the treat-and-extend regimen was introduced.^4^ This approach involves an initial loading phase of three injections, followed by extending the treatment intervals by 2 weeks at a time—up to 12, 16, or 20 weeks—if the CNV remains inactive. If reactivation occurs, the intervals are shortened by 2 weeks.^5^

The treat-and-extend regimen has gained widespread acceptance as a proactive, individualised treatment strategy and treatment intervals are extended as long as possible without disease recurrence. However, even with this approach, patients still require 7 to 10 injections annually, presenting a significant burden for both patients and the healthcare system.^5^

Multiple anti-VEGF drugs are approved for the treatment of nAMD:

Ranibizumab is a fragmented monoclonal antibody (Fab fragment) that specifically inhibits VEGF-A. Aflibercept is a fusion protein (VEGF trap) that binds both VEGF-A and PlGF.

Brolucizumab is a humanised small molecule monoclonal antibody that binds VEGF-A.

Another drug, bevacizumab, is a monoclonal antibody that directly binds and neutralises VEGF and is widely used as an off-label treatment. In response, recent pharmaceutical advancements have focused on strategies that extend the durability of intravitreal therapies. These efforts include expanding pharmacological targets beyond VEGF and increasing the dose of anti-VEGF agents. One of these strategies was introduced in 2022 with faricimab, a bispecific antibody that inhibits both VEGF and angiopoietin-2.^6^

More recently, the Food and Drug Administration and the European Medicines Agency have approved a higher dose of aflibercept, with 8 mg (four times the previous 2 mg dose) marking a significant development in nAMD treatment.^7^

The CANDELA (phase II) as well as PULSAR (phase III) trials have shown the safety and efficacy of the treatment of nAMD patients with intravitreal aflibercept with an increased dose of 8 mg giving promising outlooks for this new formulation. However, registrational trials are performed under highly controlled conditions with strict patient compliance, whereas real-world treatment often encounters lower adherence and less stringent patient selection.

To gain a deeper understanding of the mechanism of action and therapeutic effects of increased dosage of aflibercept, it is crucial to assess not only the commonly used quantitative measures such as mean central retinal thickness (CRT; mean thickness within the 1 mm ETDRS-circle (Early Treatment Diabetic Retinopathy Study) centred on the fovea) and best-corrected visual acuity (BCVA), but also a range of optical coherence tomography (OCT) biomarkers associated with nAMD. However, manually analysing these biomarkers and monitoring their changes over time, particularly in large patient cohorts, can be time-consuming, labour-intensive and prone to variability. The diverse morphological features of AMD further complicate the interpretation of these scans.

By using a previously trained deep learning-based semantic segmentation algorithm^8^, we were able to quantitatively assess the change of biomarkers such as fibrovascular pigment epithelial detachment (fvPED), intraretinal fluid (IRF), subretinal fluid (SRF) and subretinal hyperreflective material (SHRM) over time. This method offers a comprehensive evaluation of the effects of aflibercept 8 mg on disease activity in nAMD.

This retrospective real-world study aims to assess the efficacy and durability of the increased dosage of aflibercept 8 mg in patients with recalcitrant nAMD during the first four intravitreal injections by quantitatively analysing various OCT-based nAMD biomarkers.

## Methods

### Participants

This retrospective study analysed the Smart Eye Database from the Department of Ophthalmology at LMU University Hospital Munich, identifying patients who received aflibercept 8 mg for neovascular AMD between March 2024 and September 2024.

The inclusion criteria were: (i) a switch to 8 mg aflibercept following previous intravitreal therapy for nAMD with aflibercept 2 mg immediately before the switch, due to an inadequate response defined by the persistence of intraretinal or SRF despite monthly anti-VEGF injections or the inability to extend treatment intervals beyond 6 weeks (fluid recurrence after 7 weeks); (ii) completion of at least four intravitreal injections of 8 mg aflibercept; and (iii) no confounding conditions such as intraocular infection or uveitis.

Ethics approval was obtained from the Institutional Review Board of the Faculty of Medicine, LMU Munich, study ID: 20–0382, and the study adhered to the principles outlined in the Declaration of Helsinki.

It was not appropriate or possible to involve patients or the public in the design, conduct, reporting or dissemination plans of our research.

Epidemiological data collected for each patient included age, gender, date of initial nAMD diagnosis, the extent of prior intravitreal injection therapy and the date of transition to 8 mg aflibercept.

### Preoperative examinations

Pre-injection assessments included BCVA, intraocular pressure (IOP) measurement with non-contact tonometry and dilated indirect funduscopy.

Multimodal imaging was performed as needed, including spectral-domain OCT (SD-OCT) and near-infrared scanning with the Spectralis Heidelberg Retina Angiograph (HRA) + OCT system (Heidelberg Engineering) at each visit. At the time of initial diagnosis, confirmation of CNV was obtained through OCT-angiography and/or fluorescein angiography.

OCT data were collected at the first intravitreal administration of aflibercept 8 mg (v0) and the following three treatment visits (v1, v2, v3). In general, no additional loading phase was administered after switching to the increased dosage of aflibercept, that is, the treat-and-extend scheme was continued, leading to different time points of v1, v2 and v3 for every patient.

### Automated quantification of biomarkers

Biomarker segmentation was carried out using a deep learning-based semantic segmentation algorithm developed by Asani *et al* in collaboration with Helmholtz Zentrum Munich.^8^ This artificial intelligence (AI)-driven system automates the segmentation of nAMD-related biomarkers by leveraging a deep convolutional neural network, which has shown performance comparable to manual segmentation by retinal experts, as evidenced by high F1 scores. The algorithm classifies each pixel in OCT B-scans, distinguishing between various biomarkers and normal retinal tissue, based on the Consensus Nomenclature for Reporting Neovascular Age-Related Macular Degeneration, established by the American Academy of Ophthalmology (AAO).^9^

The neural network was trained on a large dataset of OCT scans confirmed by pathology and annotated by expert graders. It produces quantitative outputs using the standardised ETDRS grid, ^10^with CRT measured in micrometres and volumetric biomarkers represented in arbitrary pixel units to preserve accuracy. This approach avoids distortions that could arise from converting to the metric system, particularly from image compression. By focusing on temporal changes and value differences, this method ensures the most reliable tracking of biomarker progression over time.

### Data analysis and statistics

Data management was performed using Microsoft Excel V.16.78.3 for Mac, and statistical analyses were conducted with GraphPad Prism for macOS V.10.3.1. A significance level of p<0.05 was applied.

Pairwise comparisons of biomarker changes from v0 to v3 were conducted using the Wilcoxon matched-pairs signed-rank test. Pearson’s correlation coefficient (r) was used to assess the relationships between dependent and independent variables. Demographic data are shown as mean±SD; as no normal distribution could be shown in the case of biomarkers and visual acuity, corresponding data are reported as median and interquartile range (IQR).

## Results

### Baseline demographics

A total of 23 eyes from 21 patients with treatment-resistant nAMD were switched to aflibercept 8 mg during the study period.

Baseline demographics are detailed in table 1. The average age at switching was 78.87±7.44 years, with a gender distribution of 17 women and four men. All patients were treated with aflibercept 2 mg before being switched to a higher dosage of 8 mg aflibercept. On average, patients had received 30.70±22.54 anti-VEGF injections before switching, including 10.17±11.25 injections of ranibizumab, 17.55±16.57 injections of aflibercept 2 mg and 1.48±3.09 injections of faricimab. Six eyes (26%) had received less than 15 prior anti-VEGF injections before the switch to aflibercept 8 mg. Eight eyes (35%) were pretreated with between 15 and 30 injections, and nine eyes (39%) had already had 30 or more injections before the change of medication. The mean follow-up time was 112.17±21.02 days after the initial intravitreal administration of aflibercept 8 mg. No severe ocular complications, including intraocular inflammation or vasculitis, retinal detachment, significant increases in IOP, intraocular haemorrhages or retinal pigment epithelium tears, were reported during the study period.

**Table 1:** Baseline demographics including number of patients, number of eyes, age, gender and prior intravitreal treatment

Number of patients	21
Number of eyes	23
Mean age (years)	78.87±7.44
Gender	
Female	17
Male	4
Mean prior anti-VEGF injections	
Total (n)	30.70±22.54
Total RBZ	10.17±11.25
Total AFL	17.55±16.57
Total FAR	1.48±3.09
Anti-VEGF injections prior to switch	
Less than 15 injections	6 eyes (26%)
Between 15 and 30 injections	8 eyes (35%)
30 or more injections	9 eyes (39%)
Mean time of pretreatment	3.49±2.98 years
Mean injections per year	10.39±2.76
Mean follow-up time (days)	112.17±21.02

AFL, Aflibercept 2 mg; FAR, faricimab; RBZ, ranibizumab; VEGF, vascular endothelial growth factor.

### Biomarker changes

**Figure 1:**
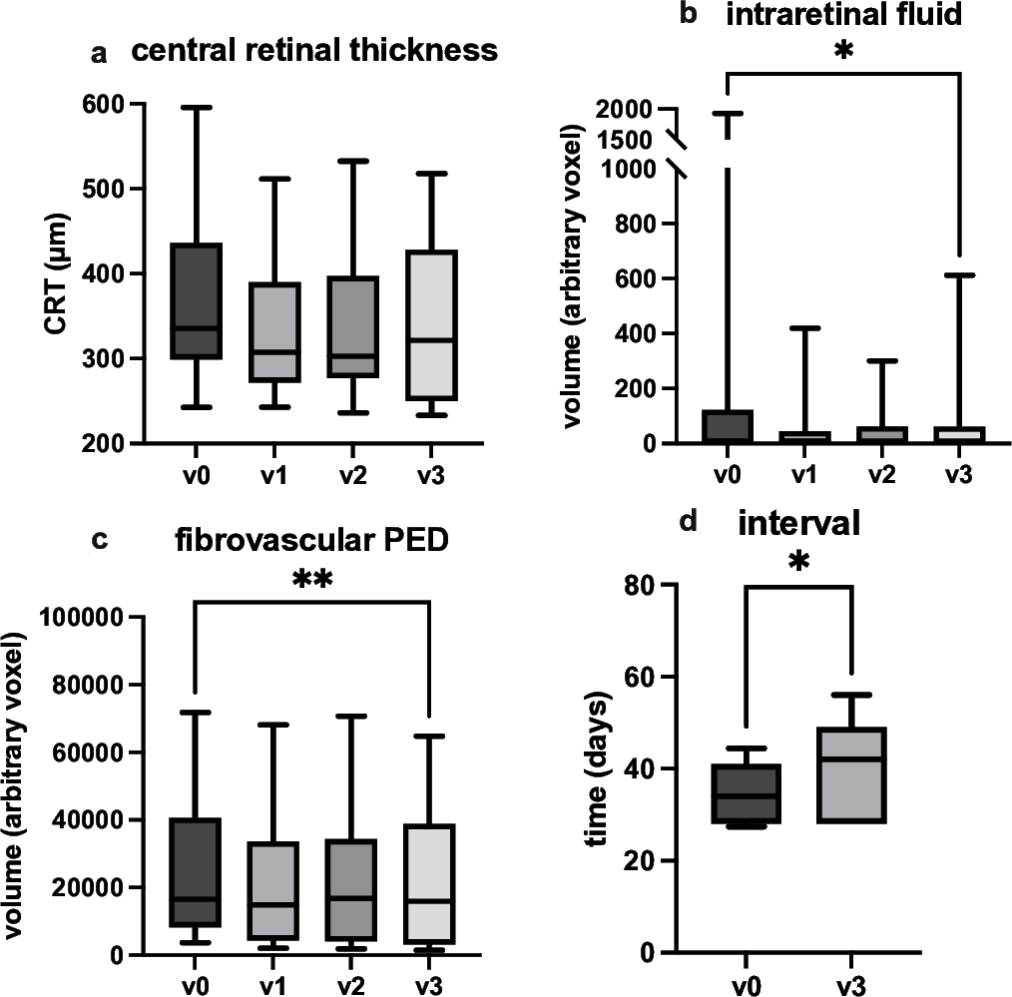
Timepoints are v0 (day of switch, first aflibercept 8 mg injection), v1 (day of second aflibercept 8 mg injection), v2 (day of third intravitreal high-dose aflibercept application) and v3 (day of fourth 8 mg aflibercept injection). p-values of pairwise comparison are indicated by asterisks (with ** as p<0.01; * as p<0.05).

At baseline, all eyes exhibited signs of MNV activity, including IRF, SRF or fvPED. Detailed biomarker changes at each visit are presented in table 2 and figure 1.

**Table 2:** Measures of BCVA, CRT, OCT biomarkers and injection interval during the study period.

	BCVA (logMAR)	CRT (µm)	SRF (voxel)	IRF (voxel)	SHRM (voxel)	fvPED (voxel)	Injection interval (days)
v0	0.2007	335.6	613	8	26	16 501	34
v1	0.2007	307.4	30	9	21	14 893	–
v2	0.2007	302.9	86	6	9	16 811	–
v3	0.2007	321.6	84	2	29	15 953	42
iqr0	0.39799	136.9	8589	122	171	32 354	13
iqr1	0.30099	118.1	2178	45	239	29 314	–
iqr2	0.39799	120.0	1795	61	95	30 180	–
iqr3	0.30099	178.1	4277	61	186	35 565	21
p value (v0–v3)	0.7297	0.0522	0.3209	**0.0198**	0.9922	**0.0015**	**0.026**

Pairwise comparison of changes from v0 to v3 (significant p-values in bold).

Time points: v0 (day of switch, first 8 mg aflibercept injection), v1 (day of second aflibercept 8 mg injection), v2 (day of third intravitreal high-dose aflibercept application) and v3 (day of fourth 8 mg aflibercept injection).

BCVA, best-corrected visual acuity; CRT, central retinal thickness; fvPED, fibrovascular pigment epithelial detachment; IRF, intraretinal fluid ; OCT, optical coherence tomography; SHRM, subretinal hyperreflective material; SRF, subretinal fluid.

CRT, as a surrogate marker for disease activity, showed no statistically significant change from 335.6 µm (IQR: 136.9 µm) at v0 to 321.6 µm (IQR: 178.1 µm) at v3. However, when analysing the different biomarkers independently, significant changes could be observed (see figure 1).

After the third intravitreal administration of aflibercept 8 mg, at v3, there was a significant reduction in IRF and fvPED compared with v0. When comparing the patient cohort at baseline (v0) to those at timepoint v3, IRF changed statistically significant from 8 (IQR: 122) voxel to 2 (IQR: 61) voxel (p=0.0198) and fvPED from 16 501 (IQR: 32354) voxel to 15 953 (IQR: 35565) voxel (p=0.0015).

A reduction of SRF could be observed from 613 (IQR: 8589) voxel to 84 (IQR: 4277) voxel; however, this change was not statistically significant (p=0.3209). SHRM showed no significant change from 26 (IQR: 171) voxel to 29 (IQR 186) (p=0.9922).

All data, along with p values from the statistical analyses, are presented in table 2. Errors are reported as interquartile ranges due to the non-normal distribution of the data.

### Treatment intervals

In general, no new loading phase was administered after switching from aflibercept 2 mg to the increased dosage of aflibercept 8 mg, that is, the treat-and-extend scheme was continued, leading to different timepoints of v1, v2 and v3 for every patient. Keeping in mind the continued prolongation of intervals due to the treat-and-extend scheme, the mean injection interval was significantly extended from 34 (IQR: 13) days before the switch at v0 to 42 (IQR: 21) days after the switch by the end of the follow-up period at v3, representing a 24% extension.

### Visual acuity

Visual acuity, measured by the LogMAR score, remained stable throughout the intravitreal treatment with high-dose aflibercept, with no statistically significant changes observed. The scores were 0.2007 (IQR: 0.39799) LogMAR at v0 (before switching), 0.2007 (IQR: 0.30099) LogMAR at v1 (after the first injection), 0.2007 (IQR: 0.39799) LogMAR at v2 (after the second injection) and 0.2007 (IQR: 0.30099) LogMAR, respectively (after the third injection); as no normal distribution is present, data are reported as median and IQR. Detailed data are provided in table 2.

### Correlation analysis

We also analysed the correlation between the change of different biomarkers and the improvement in BCVA.

We found a statistically significant correlation between the reduction of CRT from v0 to v3 and the increase in BCVA from v0 to v3 (p=0.0486) with a Pearson’s r of 0.3543. Concerning the other biomarkers analysed, no statistically significant correlation was found.

Findings are summarised in table 3.

**Table 3:** Correlation analysis between the difference of BCVA in LogMAR (from v0 to v3) and the change of the different biomarkers (from v0 to v3)

R	ΔlogMAR (v0–v3)	P
ΔCRT (v0–v3)	0.3543	**0.0486**
ΔSRF (v0–v3)	0.2907	0.0892
ΔIRF (v0–v3)	−0.1196	0.2935
ΔfvPED (v0–v3)	−0.04016	0.4278
ΔSHRM (v0–v3)	0.1295	0.2779

Significant p values in bold.

CRT, central retinal thickness; fvPED, fibrovascular pigment epithelial detachment; IRF, intraretinal fluid; SHRM, subretinal hyper-reflective material; SRF, subretinal fluid.

## Discussion

To our knowledge, this study is the first to describe the quantitative change of OCT biomarkers during real-world application of high-dose 8 mg aflibercept in patients with recalcitrant nAMD previously treated with 2 mg aflibercept.

In literature, opinions differ on whether increasing the molar dose of aflibercept significantly improves its efficacy and durability. The Phase II CANDELA trial found no superiority of 8 mg aflibercept over 2 mg in terms of efficacy, though trends towards anatomical benefits were noted.^11^ In contrast, the Phase III PULSAR trial demonstrated non-inferiority of 8 mg aflibercept compared with 2 mg, with significantly extended durability. However, the PULSAR trial’s retreatment criteria, which required both the presence of fluid on OCT and a loss of at least five ETDRS letters in visual acuity, differed from the standard care, complicating the assessment of treatment durability compared with 2 mg aflibercept.^12^

It may be useful to look at evidence from earlier trials investigating increased molar doses of anti-VEGF agents. For instance, the HARBOR trial, which studied 2 mg ranibizumab (four times the standard dose), did not show superiority in visual acuity. However, HARBOR was designed to demonstrate superiority in visual outcomes, whereas most contemporary trials focus on achieving non-inferiority with fewer injections.^13^ This was demonstrated in the TENAYA and LUCERNE trials, which explored faricimab, a bispecific antibody targeting both VEGF and Angiopoietin-2, allowing for extended dosing intervals.^6^

Smaller studies have also investigated off-label increases in aflibercept dosing. For example, You *et al* treated recalcitrant nAMD with 4 mg aflibercept (double the standard dose) and found significant anatomical improvements, with 45% of eyes showing at least one-line improvement in visual acuity after 1 month.^14^ Similar results were seen with 2 mg and 3 mg aflibercept in other studies.^15 16^ These findings suggest that increased dosing may lead to improved anatomical and functional outcomes, highlighting the need for comparative trials between 2 mg and 8 mg aflibercept, particularly for patients with recalcitrant nAMD who show suboptimal responses to established treatment options.

In this study, a deep learning-based semantic segmentation algorithm was used to track changes in key biomarkers over time. This algorithm enabled automated and precise quantification of biomarkers from OCT scans, enhancing both the accuracy and efficiency of the analysis.

The findings revealed that high-dose aflibercept significantly increased the treatment interval in a treat-and-extend regimen by at least maintaining the amount of key disease activity biomarkers. Even though no significant change from v0 to v3 could be found for the well-established surrogate marker of CRT, analysis of the single biomarkers revealed a significant reduction in IRF and fvPED; SRF also showed a reduction, but not statistically significant. As especially IRF is connected with a worse outcome during AMD treatment compared with SRF,^17^ patients could particularly benefit from the fact that high-dose aflibercept leads to a reduction in this more aggressive retinal fluid in the long run.

It is important to highlight here that we were particularly treating patients who have already received various other anti-VEGF drugs and have shown an inadequate response to therapy or repeated recurrence after frustrating treatment extensions. In these patients, we were able to stabilise the disease activity by switching to aflibercept 8 mg, thereby extending the intervals and thus also the treatment burden for the patient. Notably, injection intervals were extended by an average of 24%, demonstrating the potential to lessen the treatment burden in this difficult-to-treat patient group. Using correlation analysis, we found that a reduction in CRT was connected with an increase in BCVA after switching to high-dose aflibercept.

The use of artificial intelligence for automated biomarker segmentation presents a promising path for more efficient, objective and scalable monitoring of disease progression in clinical practice. Future studies should aim to extend follow-up beyond 3 months to evaluate the long-term efficacy and safety of high-dose aflibercept, especially in real-world settings. Larger patient groups analysed will lead to higher statistical power and reveal a significant improvement in other biomarker characteristics. Additionally, an important question to address is how AI-driven analysis can be integrated into routine clinical workflows to enhance personalised treatment strategies and optimise outcomes for patients with nAMD. The combination of innovative therapeutic options with advanced AI tools holds great potential for improving the management of complex retinal diseases and enhancing patient quality of life.

## Data Availability

Data are available upon reasonable request.
